# Drought response in Arabidopsis displays synergistic coordination between stems and leaves

**DOI:** 10.1093/jxb/erac446

**Published:** 2022-11-09

**Authors:** Ajaree Thonglim, Giovanni Bortolami, Sylvain Delzon, Maximilian Larter, Remko Offringa, Joost J B Keurentjes, Erik Smets, Salma Balazadeh, Frederic Lens

**Affiliations:** Naturalis Biodiversity Center, Research Group Functional Traits, PO Box 9517, 2300 RA Leiden, The Netherlands; Naturalis Biodiversity Center, Research Group Functional Traits, PO Box 9517, 2300 RA Leiden, The Netherlands; BIOGECO INRA, Université Bordeaux, 33615 Pessac, France; BIOGECO INRA, Université Bordeaux, 33615 Pessac, France; Leiden University, Institute of Biology Leiden, Plant Developmental Genetics, Sylviusweg 72, 2333 BE Leiden, The Netherlands; Laboratory of Genetics, Wageningen University, Droevendaalsesteeg 1, 6708 PB Wageningen, The Netherlands; Naturalis Biodiversity Center, Research Group Functional Traits, PO Box 9517, 2300 RA Leiden, The Netherlands; Leiden University, Institute of Biology Leiden, Plant Sciences, Sylviusweg 72, 2333 BE Leiden, The Netherlands; Leiden University, Institute of Biology Leiden, Molecular Plant Stress Biology, Sylviusweg 72, 2333 BE Leiden, The Netherlands; Naturalis Biodiversity Center, Research Group Functional Traits, PO Box 9517, 2300 RA Leiden, The Netherlands; Leiden University, Institute of Biology Leiden, Plant Sciences, Sylviusweg 72, 2333 BE Leiden, The Netherlands; Hong Kong Baptist University

**Keywords:** *Arabidopsis thaliana*, chlorophyll content, drought response, embolism resistance, gene expression, intervessel pit membrane thickness, stem anatomy, stomatal control

## Abstract

The synergy between drought-responsive traits across different organs is crucial in the whole-plant mechanism influencing drought resilience. These organ interactions, however, are poorly understood, limiting our understanding of drought response strategies at the whole-plant level. Therefore, we need more integrative studies, especially on herbaceous species that represent many important food crops but remain underexplored in their drought response. We investigated inflorescence stems and rosette leaves of six *Arabidopsis thaliana* genotypes with contrasting drought tolerance, and combined anatomical observations with hydraulic measurements and gene expression studies to assess differences in drought response. The *soc1ful* double mutant was the most drought-tolerant genotype based on its synergistic combination of low stomatal conductance, largest stomatal safety margin, more stable leaf water potential during non-watering, reduced transcript levels of drought stress marker genes, and reduced loss of chlorophyll content in leaves, in combination with stems showing the highest embolism resistance, most pronounced lignification, and thickest intervessel pit membranes. In contrast, the most sensitive Cvi ecotype shows the opposite extreme of the same set of traits. The remaining four genotypes show variations in this drought syndrome. Our results reveal that anatomical, ecophysiological, and molecular adaptations across organs are intertwined, and multiple (differentially combined) strategies can be applied to acquire a certain level of drought tolerance.

## Teaser

Drought response among *Arabidopsis thaliana*genotypes is clearly different and determined by the interplay between stem (embolism resistance, xylem anatomy) and leaf (stomatal control, chlorophyll loss, gene regulation) traits.

## Introduction

The increasing intensity and frequency of drought episodes are becoming major threats to current and future agricultural productivity around the globe. Even the countries that had not experienced drought stress during the last decades are now impacted by drought (Corso *et al.*, 2020; Gleason *et al.*, 2022). One of the major problems that plants experience when they are facing severe drought is that detrimental levels of drought-induced gas bubbles (embolisms) in the xylem sap generate massive obstruction of the root to shoot water transport (Sperry and Tyree, 1988; Tyree and Zimmermann, 2002; Cochard, 2006; Choat *et al.*, 2012; Venturas *et al.*, 2017; Johnson *et al.*, 2022), which happens after stomata are closed (Martin-StPaul *et al.*, 2017). Stomatal closure may result in reduced photosynthetic productivity, growth rate, and reproduction, and under conditions of intense and prolonged drought may eventually cause desiccation and dieback of tissues (Mantova *et al.*, 2022), organs, and entire plants (Davis *et al.*, 2002; Venturas *et al.*, 2016; Pratt *et al.*, 2020; Brodribb *et al.*, 2021). Lethal levels of embolism, from which plants are unable to recover, are thought to be reached when the hydraulic conductivity is reduced to ~88% of its maximum conductance (*P*_88_) (Urli *et al.*, 2013; Li *et al.*, 2015; but see Hammond *et al.*, 2019; Johnson *et al.*, 2021), although there are probably more accurate thresholds to drought-induced mortality than *P*_88_ (Mantova *et al.*, 2021, 2022). Due to the implications of dramatic levels of drought-induced embolism on productivity, tissue death, and long-term survival, there is increasing evidence that natural selection has shaped the hydraulic systems of plants to minimize embolism occurrence and water potential loss during periods of water shortage (Lens *et al.*, 2022). This can be made possible when many drought-related traits from different organs act in concert (Dayer *et al.*, 2022).

As an example, angiosperms can build more resistant xylem by modifying a whole array of xylem anatomical adaptations to prevent the spread of embolisms, such as fine-scale modifications of pits in vessel walls allowing lateral transport of water and gas between adjacent vessels (Lens *et al.*, 2011; Li *et al.*, 2016; Kaack *et al.*, 2019, 2021; Levionnois *et al.*, 2021), or increased levels of lignification (Lens *et al.*, 2013, 2016; Thonglim *et al.*, 2020). In addition, plants can also delay xylem sap pressures from reaching critical embolism thresholds throughout the whole-plant body by producing the stress hormone abscisic acid (ABA) that induces stomatal closure in the leaves very rapidly at the onset of drought, well before embolism events start to exponentially increase (Brodribb *et al.*, 2017; Martin-StPaul *et al.*, 2017; Buckley, 2019; Creek *et al.*, 2020). Consequently, stomatal closure is one of the primary responses that helps restrict water loss, which safeguards the water potential in the leaves and buffers the negative pressure in xylem sap (Brodribb *et al.*, 2017; Martínez-Vilalta and Garcia-Forner, 2017; Martin-StPaul *et al.*, 2017; Knipfer *et al.*, 2020). The regulation of water potential in leaves during drought is crucial because it influences plant metabolic processes. However, declining transpiration rates reduce not only water loss but also carbon uptake, leading to decreased photosynthetic activity, which ultimately may lead to carbon starvation when stomata remain closed for a long time (McDowell *et al.*, 2008). In other words, the interplay between embolism resistance inside the plant’s xylem and the onset and duration of stomatal closure at the level of leaves will determine how long leaves can remain metabolically active without risk of detrimental levels of drought-induced embolism (Allen *et al.*, 2010; Choat *et al.*, 2012; Mitchell *et al.*, 2013; Brodribb *et al.*, 2017; Martínez-Vilalta and Garcia-Forner, 2017; Buckley, 2019; Creek *et al.*, 2020; Limousin *et al.*, 2022). Accordingly, the stomatal safety margin (SSM), which can be defined as the difference between the water potential at stomatal closure (Ψ*g*_s90_) and the pressure inducing 50% loss of hydraulic conductance (*P*_50_) is physiologically more important to estimate a plant’s ability to cope with massive levels of drought-induced embolism than only *P*_50_ (Sperry and Tyree, 1988; Meinzer *et al.*, 2009; Anderegg *et al.*, 2016; Martin-StPaul *et al.*, 2017; Creek *et al.*, 2020; Dayer *et al.*, 2020; Skelton *et al.*, 2021). It is widely accepted that species with a narrower safety margin are operating more closely to their hydraulic threshold, while species that have a wider safety margin have a lower risk of facing a detrimental level of drought-induced embolism (Choat *et al.*, 2012; Anderegg *et al.*, 2016; Martin-StPaul *et al.*, 2017; Eller *et al.*, 2018; Creek *et al.*, 2020; Oliveira *et al.*, 2021; Skelton *et al.*, 2021).

It is clear that anatomical and physiological traits need to be intertwined within and among organs, but the molecular mechanisms cross-linking different pathways remain elusive. For instance, there is increasing evidence from gene expression studies confirming the positive correlation between lignification and drought resilience in a whole range of species (Tu *et al.*, 2020; Xu *et al.*, 2020; Wen *et al.*, 2021; Yan *et al.*, 2021; Hou *et al.*, 2022; Li *et al.*, 2022). Regarding drought responses in plants, the ABA-mediated signalling pathway is probably the best-known pathway at the molecular level. ABA regulates the expression of stress-responsive genes via transcription factors (Bauerle *et al.*, 2004; Cutler *et al.*, 2010; Bauer *et al.*, 2013; Dodd, 2013; Mehrotra *et al.*, 2014; Chen *et al.*, 2020). Once ABA is accumulated, it regulates ABA-responsive genes via the *cis*-element called ABRE (ABA-responsive element) in their promoter regions using AREB (ABRE binding) transcription factors (Choi *et al.*, 2000; Uno *et al.*, 2000; Yoshida *et al.*, 2015; Chen *et al.*, 2020). In Arabidopsis, *AREB1* is mainly expressed in vegetative tissues and up-regulated during drought (Yoshida *et al.*, 2010; Fujita *et al.*, 2011, 2013; Singh and Laxmi, 2015; Chen *et al.*, 2020). Other drought-responsive genes are regulated by dehydration-responsive element-binding (DREB) proteins through an ABA-independent pathway (Bartels and Sunkar, 2005; Sakuma *et al.*, 2006; Song *et al.*, 2018). For example, DREB2 transcription factors are induced by dehydration and are involved in gene transcription under water shortage (Agarwal *et al.*, 2006; Song *et al.*, 2018). Interestingly, many stress-inducible genes contain both ABREs and DREs in their promoter regions, such as *Responsive to Desiccation 29* (*RD29*) (Shinozaki and Yamaguchi-Shinozaki, 2007). Hence, gene expression of drought-responsive genes occurs via ABA-dependent and/or ABA-independent signal transduction pathways (Umezawa *et al.*, 2010; Rushton *et al.*, 2012; Song *et al.*, 2016), and allows us to evaluate the expression of drought-responsive genes during a drought experiment with a simultaneous assessment of physiological and anatomical traits involved in drought tolerance.

Most studies investigating drought-induced embolism in plants have been focusing on trees, while herbaceous plants have been largely ignored despite their importance as crops and food sources for humans and animals (Brodribb and Hill, 1999; Stiller and Sperry, 2002; Holloway-Phillips and Brodribb, 2011; Choat *et al.*, 2012; Ahmad, 2016; Lens *et al.*, 2016; Volaire *et al.*, 2018). In our previous study on the herbaceous model species, *Arabidopsis thaliana*, including genotypes with contrasting levels of embolism resistance and lignification in the inflorescence stems (Thonglim *et al.*, 2020), we found that the more lignified genotypes are more resistant to embolism and have thicker intervessel pit membranes. Surprisingly, in most structure–function studies published so far, the drought response is only partly observed due to methodological and time constraints. For instance, resistance to embolism in branches/twigs is often recorded in xylem physiological studies (e.g. Choat *et al.*, 2012; Anderegg *et al.*, 2016), and less frequently integrated with leaf *P*_50_ data (e.g. Cochard *et al.*, 2004; Klepsch *et al.*, 2018; Skelton *et al.*, 2019; Levionnois *et al.*, 2021) and/or root *P*_50_ data (e.g. Rodriguez-Dominguez *et al.*, 2018), and sometimes linked with other leaf physiological traits such as stomatal conductance (*g*_s_) and water potential (e.g. Brodribb *et al.*, 2003; Li *et al.*, 2015; Cardoso *et al.*, 2018; Charrier *et al.*, 2018; Creek *et al.*, 2020; Chen *et al.*, 2021). Only occasionally are detailed hydraulic measurements in stems, leaves, and/or roots complemented with detailed anatomical traits on intervessel pits (Guan *et al.*, 2022). Other papers only focus on the molecular pathway and gene regulation during drought (e.g. Bhargava and Sawant, 2013; Pandey *et al.*, 2013; Janiak *et al.*, 2016; Ebrahimian-Motlagh *et al.*, 2017; Thirumalaikumar *et al.*, 2018; Roca-Paixão *et al.*, 2019; Zhang *et al.*, 2019), while publications that integrate gene function with xylem physiology are scarce (e.g. Kitin *et al.*, 2010; Lamarque *et al.*, 2020). Integration of drought-related traits across organs in structure–function studies and intensive collaboration among plant anatomists, xylem physiologists, and molecular biologists will help us to make considerable progress in a holistic understanding of drought response at the whole-plant level. To contribute to that whole-plant approach, we measured hydraulic traits in stems and leaves during a drought experiment, combined with detailed stem anatomical measurements and an assessment of transcript levels of drought stress marker genes across Arabidopsis genotypes (two transgenic lines and four natural accessions).

In this study, we investigate the following two questions. (i) Is there a coupling between drought-related stem (anatomy, *P*_50_) and leaf traits (stomatal regulation, leaf water potential, expression of drought marker genes) among Arabidopsis genotypes? (ii) Can these genotypes use different combinations of drought-response traits to reach a certain level of drought tolerance? To answer these questions, we investigated six genotypes with marked differences in embolism resistance and lignification of the inflorescence stems. We examined the detailed stem anatomical traits and hydraulic traits (stem *P*_50_) of each genotype and quantified the drought response for all six genotypes using a drought experiment, during which we measured *g*_s_ and leaf water potential (Ψ_l_), allowing us to calculate the SSM (as defined by Ψ*g*_s90_ minus *P*_50_). In addition, we compared the expression of four drought-responsive genes from the ABA-(in)dependent (*ABI2*, *AREB1, RD29A*, and *DREB2A*) pathways from the rosette leaves at the end of the drought experiment to validate the level of drought stress among the six genotypes. By integrating all traits mentioned above, we want to assess how anatomical and ecophysiological traits across organs are intertwined to acquire a certain level of drought tolerance, and how these traits relate to the drought stress level at the end of the drought experiment based on a limited number of drought stress marker genes.

## Materials and methods

### Plant material

In addition to the four *A. thaliana* genotypes with contrasting levels of stem *P*_50_ and stem lignification, we studied before the ecotypes Columbia-0 (Col-0; wild type with intermediate stem lignification), Shadarah (Sha; wild type with a higher level of stem lignification), Cape Verde Islands (Cvi; least lignified wild type), and the double loss-of-function mutant *SUPPRESSOR OF OVEREXPRESSION OF CONSTANS 1* and *FRUITFULL* (*soc1ful*; most lignified genotype) (see Thonglim *et al.*, 2020); we added one more wild type [Kelsterbach-4 Kel-4)] and a *p35S:AHL15* line (*AHL15* overexpression) in the Col-0 background (Rahimi *et al.*, 2022). The two additional genotypes were selected based on their inflorescence length (at least 27 cm required for the centrifuge method used to estimate embolism resistance measurements) and their increased lignification in the basal parts of the inflorescence stem, respectively (Supplementary Fig. S1A, B, G, H). Indeed, Kel-4, an early flowering ecotype from Germany, shows a relatively high proportion of lignification at the base of the inflorescence stem (Ak, 2020), and has been reported to be more drought tolerant compared with many other wild-type accessions (Bac-Molenaar *et al.*, 2016; Kooke *et al.*, 2016). The *AT-HOOK MOTIF CONTAINING NUCLEAR LOCALIZED 15* (*AHL15*) gene has been found to suppress axillary meristem maturation, and its overexpression extends plant longevity (Karami *et al.*, 2021), and promotes secondary growth in the inflorescence stem to a similar extent as the *soc1ful* mutant (Rahimi *et al.*, 2022).

### Growing conditions

The plants were grown at the Institute of Biology Leiden (Leiden University, The Netherlands) under the same controlled conditions as in Thonglim *et al.* (2020) to ensure comparable datasets. Briefly, we germinated the two additional genotypes from seeds directly into a mixture of soil and sand (4.5:1). After 10 d of germination, the healthy seedlings were transferred into pots. Plants were grown in a controlled growth chamber with the following parameters: 20 °C temperature during the day and 17 °C temperature at night, 70% relative humidity, and 16 h photoperiod condition with 100 µmol m^−2^ s^−1^ light intensity. Sampling was synchronized based on differences in flowering time and subsequent inflorescence development. To synchronize flowering, *p35S:AHL15* plants were planted earlier (harvesting inflorescence stems 85 d after sowing). The Kel-4 individuals were planted slightly later (harvesting inflorescence stems 65 d after sowing) (Supplementary Fig. S1A, B).

### Drought experiment

A drought experiment was performed to assess the link between the anatomical and hydraulic traits and investigate the differences in drought tolerance across the six *A. thaliana* genotypes studied. The six genotypes were selected based on a previous screening of drought tolerance and the differences in stem lignification (Melzer *et al.*, 2008; Bac-Molenaar *et al.*, 2016; Thoen *et al.*, 2017; Thonglim *et al.*, 2020). The seeds of each genotype were directly sown in 6 cm pots (27 g) with the same amount of soil and sand mixture (4.5:1) at different times to synchronize flowering. The weight of the pot with dry and saturated soil was controlled (807 g and 1097 g, respectively). The pots were kept in a growth-controlled chamber under the same conditions as the individuals grown for stem *P*_50_ measurements. After germination, when seedings were 10 d old, they were thinned to one healthy seeding per pot and remained well watered. We equally divided 30 individuals of each genotype into a control and a drought batch during the experiment. The control plants were well irrigated every day to keep the soil constantly hydrated (Ψ_l_ was around –0.5 MPa to –0.6 MPa). The drought batch was subjected to water deficit by completely withholding watering for 3 weeks (Ψ_l_ values ranged between –1.85 MPa to –3.4 MPa among genotypes), starting 1 week before all the genotypes began to flower. When most genotypes started developing an inflorescence stem (7 d after watering was stopped), drought measurements were initiated. Rosette leaves were harvested on the last day of the drought experiment (depending on the water potential and phenotype), immediately frozen io liquid nitrogen, and stored in a –80 °C freezer for further gene expression and chlorophyll analyses.

We initially intended to have three biological replicates per genotype. However, during sample preparation, some tubes containing ground leaf material popped open in the freezer. We assume that some liquid nitrogen used for grinding the samples was still left in the tubes, causing several closed tubes to burst open and potentially contaminate the other open tubes containing different genotypes. We opted to discard all the open tubes due to potential contamination, and use only the closed tubes. We were able to still use three biological replicates for Cvi, Sha, and *soc1ful*, but only two for Col-0, Kel-4, and *p35S:AHL15*. For the latter genotypes, we included two biological and two technical replicates.

### Chlorophyll content

Chlorophyll content was determined based on three biological replications for Cvi, Sha, and *soc1ful*, and four replicates (two biological and two technical) for Col-0, Kel-4, and *p35S:AHL15*, using the 80% acetone method (Porra *et al.*, 1989). Ground leaf samples of ~0.5 mg were transferred into 1.5 ml tubes containing 1 ml of 80% acetone. The mixtures were gently vibrated using a vortex to extract chlorophyll, and centrifuged at 1000 *g* for 5 min to remove debris. The supernatants (800 µl) were then transferred to UV-transparent microplates. The absorbance was measured at 647 nm (*A*_647_), 664 nm (*A*_664_), and 750 nm (*A*_750_) using the DMF-chl conc._YU program. Chl *a* and *b* contents (µg Chl ml^–1^) in the extract were calculated with the following formulas:


$$\text{Chl }a\;=\left(12.25\times \left(A_{664}-A_{750}\right)-2.85\times \left(A_{647}-A_{750}\right)\right)/0.29$$

$$\text{Chl }b=\left(20.31\times \left(A_{647}-A_{750}\right)-4.91\times \left(A_{664}-A_{750}\right)\right)/0.29$$

$$\text{Total Chl }\left(a+b\right)=\left(17.76\times \left(A_{647}-A_{750}\right)+7.34\times \left(A_{664}-A_{750}\right)\right)/0.2$$

**RNA isolation and qRT–PCR**

Total RNA was extracted using the RNeasy Plant Mini kit (Qiagen, Hilden, Germany). Synthesis of cDNA, quantitative reverse transcription–PCR (qRT–PCR) using SYBR Green, and data analysis were performed as previously described (Balazadeh *et al.*, 2008). Gene expression was normalized with two reference genes (*ACTIN2* and *GADPH).* qRT–PCR primers were designed using QuantPrime (www.quantprime.de) (Arvidsson *et al.*, 2008). Primer sequences are given in Supplementary Table S1. Experiments were conducted in three biological replications for Cvi, Sha, and *soc1ful*, and two biological replicates with two technical replicates for Col-0, Kel-4, and *p35S:AHL15*.

### Leaf water potential (Ψ_l_) and stomatal conductance (*g*_s_)

After 7 d of water deficit (i.e. the time required to dehydrate the moisturized soil in the pots of the drought batch), Ψ_l_ was measured in both control and drought batches every day during the drought period until harvesting (15–17 d). The daily measurements were carried out using three mature leaves (one from control and two from drought treatment) for each method. Before the measurements, the leaves were covered with aluminium foil for 30 min. Subsequently, leaf discs were cut from the bagged leaves and placed in the PSYPRO leaf water potential system (Wescor, Inc., Logan, UT, USA) to measure the leaf water potential. At the same time, *g*_s_ (mmol H_2_O m^−2^ s^−1^) was measured on single mature rosette leaves that were close to the leaves used for water potential measurements, using an SC-1 leaf porometer (METER Group, Pullman, WA, USA) that was calibrated every other day. The *g*_s_ was measured using Auto Mode configuration with desiccant. *g*_s_, depending on leaf water potential, was fit according to the following sigmoid function for each genotype using the NLIN procedure in SAS:


$$g_{s}=g_{sm}÷\left[1+exp\left(S\times \left(\Psi -\Psi gs_{50}\right)\right)\right]$$

*g*_sm_ is the maximal stomatal conductance for Ψ_**l**_=0, S the slope of the curve, and Ψ*g*_s50_ the water potential inducing 50% stomatal closure. We then estimated the water potential inducing 90% of the stomatal closure (Ψ*g*_s90_).

### Stomatal safety sargin (SSM)

The SSM was defined as the difference between the leaf water potential at 90% stomatal closure (Martin-StPaul *et al.*, 2017) calculated from the fitted curve (Ψ*g*_s90_) and the water potential at 50% loss of stem conductivity (*P*_50_):


$$SSM=\Psi_{gs90}-P_{50}$$

**Generating vulnerability curves (VCs) in stems**

#### Sample preparation of inflorescence stems

All individuals (80 individuals per genotype) were harvested at the Institute of Biology Leiden with roots, leaves, and flowers still attached and immediately wrapped in wet tissue papers. They were then enclosed in plastic bags to avoid dehydration during the shipment to the PHENOBOIS platform (INRAE, University of Bordeaux, France), where the Cavitron centrifuge measurements were performed. Before the Cavitron measurements, the roots were cut off at the basal part of inflorescence stems and trimmed on both sides, obtaining a stem segment of 27 cm in length that matches a standard Cavitron rotor. The length of the stem segments exceeds by far the maximum vessel length of Col-0, reaching only 4 cm according to Tixier *et al.* (2013) to avoid potential open-vessel artefacts (Cochard *et al.*, 2013). Next, all siliques, leaves, and flowers were removed underwater immediately before placing the inflorescence stems in the Cavitron rotor (7–9 stem segments per VC).

Xylem vulnerability to embolism was evaluated using the Cavitron method, a custom-built centrifuge that allows measuring the water flow through the inflorescence stems while spinning them to create a negative pressure in the middle part of the stem segments (Cochard, 2002; Cochard *et al.*, 2005, 2013). The negative pressure was gradually increased in each spinning step, as described in Thonglim *et al.* (2020). The degree of embolism in the xylem segment was quantified as the percentage loss of conductivity (PLC), calculated as follows:


$$PLC=100\times \left(1-\left(K/K_{max}\right)\right)$$

where *K*_max_ (m^2^ MPa^−1^ s^−1^) is the maximum hydraulic conductivity which was calculated when stem segments were fully functioning (no embolism) at low spinning speed (near 0 MPa), and *K* is the decreased hydraulic conductivity due to embolisms. The extent of embolism formation at every rotation speed was measured using the Cavisoft software (Cavisoft v1.5, University of Bordeaux, France). We fitted the data points to reconstruct the VCs using a sigmoid function based on the NLIN procedure in SAS 9.4 (SAS Institute, Cary, NC, USA) (Pammenter and Van der Willigen, 1998):


$$PLC=100÷\left[1+exp\left(\frac{S}{25}\times \left(P-P_{50}\right)\right)\right]$$

where *P* is the xylem pressure used at each rotation step, *P*_50_ is xylem pressure inducing 50% loss of hydraulic conductivity, and S (MPa^−1^) is the slope of the VC at *P*_50_.

Due to the low hydraulic conductivity of Arabidopsis, we measured vulnerability to embolism of 7–9 inflorescence stems to generate one vulnerability curve. Eight VCs were constructed for each genotype.

### Stem anatomy

Three stems from three representative VCs per genotype (nine stems per genotype) were randomly selected for light microscopy (LM) observations and one stem per VC from three VCs (three individuals per genotype) for TEM observations (Supplementary Fig. S1C, D). Both basal and central parts of the 27 cm inflorescence stem segments were sectioned because they differ in the amount of lignification (Supplementary Fig. S1E–H). We, however, invested more time in measuring trait data from the middle part than in the basal segment because that is the region where the negative pressures were applied during the Cavitron experiments, allowing us to accurately link the anatomical traits with embolism resistance (*P*_50_). The anatomical traits are represented in Supplementary Table S2. ImageJ (National Institutes of Health, Bethesda, MD, USA) was used, and the guidance of Scholz *et al.* (2013a) was followed to measure the anatomical features in digital images from both LM and TEM observations.

#### Light microscopy

Inflorescence stems were cut into 1 cm long pieces and submerged in 70% ethanol. The samples were then gradually infiltrated in LR-white resin (Hamann *et al.*, 2011). After embedding in LR-white, specimens were sectioned with a rotary microtome (Leica RM 2265, Leica, Eisenmark, Wetzlar, Germany) with disposable tungsten carbon blades at 4 µm thickness. Next, the sections were heat-fixed onto the slides, stained with 1% (w/v) toluidine blue (VWR Chemicals BDH®, Radnor, PA, USA), and mounted with DPX new-100579 mounting medium (Merck Chemicals, Amsterdam, the Netherlands). Finally, various anatomical traits (Supplementary Table S2) were observed using a Leica DM2500 light microscope equipped with a Leica DFC-425 digital camera (Leica microscopes, Wetzlar, Germany).

#### TEM

The middle parts of inflorescence stem segments were collected immediately after Cavitron measurements and fixed in Karnovsky’s fixative (Karnovsky, 1965). Subsequently, the samples were washed in 0.1 M cacodylate buffer and post-fixed with 1% buffered osmium tetroxide. The samples were then prepared for semi-thin and ultra-thin sectioning according to the protocol described in Thonglim *et al.* (2020), and were observed with a JEM-1400 Plus TEM (JEOL, Tokyo, Japan) with an 11 megapixel digital camera (Quemesa, Olympus). TEM observations were conducted to measure the intervessel pit membrane thickness and the pit chamber depth (Supplementary Table S2).

### Statistical analysis

R version 3.6.3 in R Studio version 1.2.5033 was used for the statistical analyses of all traits studied, of which all the differences were considered significant when the *P*-value was <0.05. First, general linear models with a Newman–Keuls post-hoc test were used to check the differences in embolism resistance (*P*_50_, *P*_12_, and *P*_88_), anatomical features, leaf physiological traits, chlorophyll content, and gene expression among Arabidopsis genotypes studied. Then, multiple linear regression was applied to assess the anatomical traits (predictive variables) that explain the differences in embolism resistance (responsive variables, including *P*_50_, *P*_12_, and *P*_88_). The collinearity between variables was firstly checked to select the predictors. Then, the ‘step’ function (stats package; R Core Team, 2016) was applied to achieve the most parsimonious linear regression model based on the least Akaike information criterion (AIC). Subsequently, the model’s residuals, heteroscedasticity, skewness and kurtosis, and variance inflation factor (VIF) were checked. Once we obtained the best model, the relative importance of each explanatory variable was analysed to assess the variable that explains the best *P*_50_. Pearson’s correlation was applied to plot the relationship between *P*_50_ and predictive variables and leaf physiological traits, and among the variables. Lastly, we investigated whether the different Arabidopsis genotypes presented different *g*_s_ in well-watered control conditions using a generalized linear mixed model with the accession as a fixed effect, with the GLIMMIX procedure in SAS software (SAS 9.4; SAS Institute).

### Gene codes

Arabidopsis gene codes are: *ACTIN2*, *AT3G18780*; *GAPDH*, *AT1G13440*; *RD29A*, *AT5G52310*; *ABI2*, *AT5G57050*; *AREB1*, *AT1G45249*; and *DREB2A*, *AT5G05410.*

## Results

### Drought-response phenotyping, chlorophyll content, and expression of drought-responsive genes in the basal rosette leaves

**Fig. 1.**
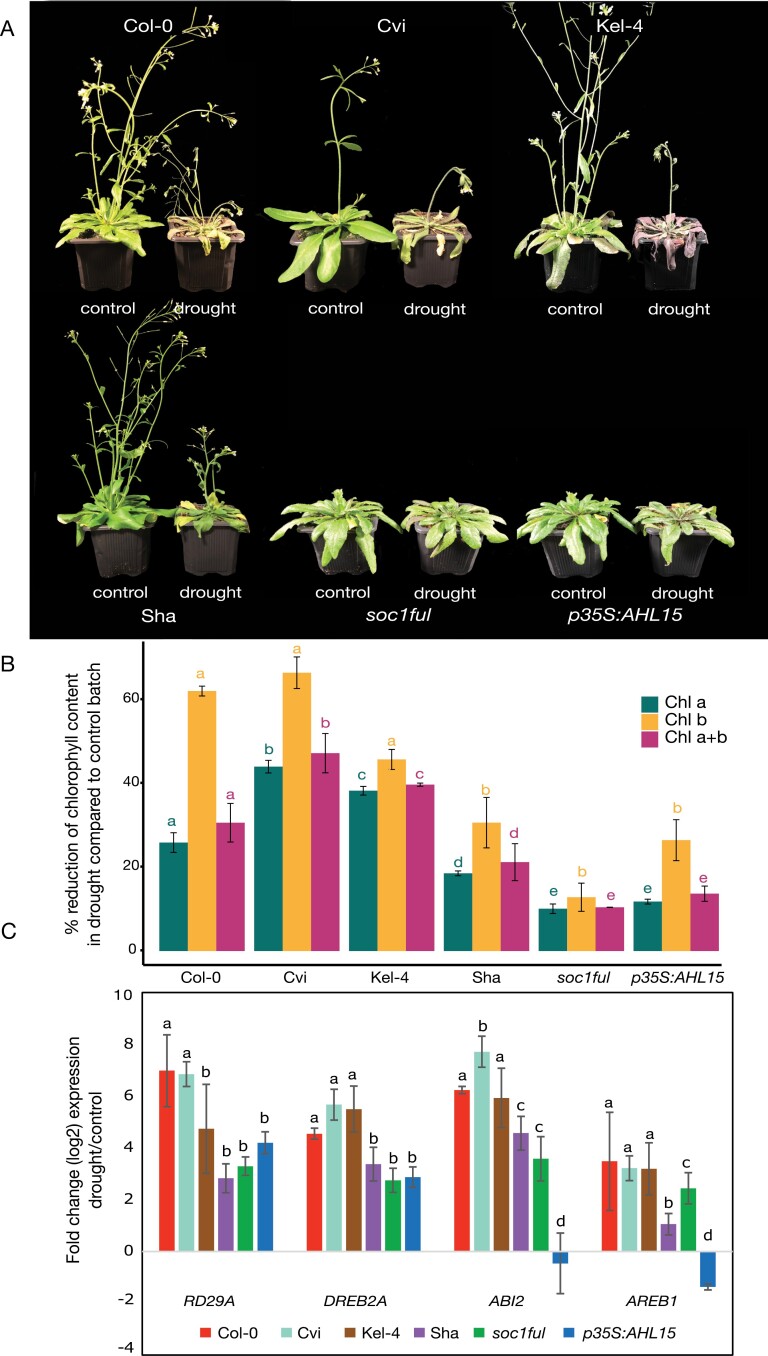
(A) Phenotypic variation in response to drought. The phenotype of six Arabidopsis genotypes subjected to drought, by water withholding at the end of a 3 week period, and their untreated counterparts. (B) The variation in chlorophyll contents (Chl *a*, Chl *b*, and Chl *a*+*b*) among genotypes studied. The *y*-axis represents the percentage reduction of chlorophyll content in drought compared with the well-watered control batch. (C) qRT–PCR analysis of the expression of selected drought-responsive genes (*RD29A*, *DREB2A*, *ABI2*, and *AREB1*) across six Arabidopsis genotypes. The *y*-axis represents the log2 fold change of the gene expression between drought and control conditions. The genes are significantly less up-regulated by drought in Sha, *p35S:AHL15*, and *soc1ful* plants. A Newman–Keuls post-hoc test was performed, showing the differences in chlorophyll reduction and gene expression level between each genotype. Different letters indicate significant differences in means of replications among genotypes; *P*-value <0.05. The error bars show the SEs based on three biological replications for Cvi, Sha, and *soc1ful*, and two biological and two technical replications for Col-0, Kel-4, and *p35S:AHL15*.

After 3 weeks of non-watering, we found differences in phenotypes of the drought-treated batch compared with the well-watered controls. The *soc1ful* mutant and the *p35S:AHL15* overexpression line were least affected by drought based on the rosette phenotype (less wilting of leaves, less reduction of rosette size) and the small reduction of chlorophyll content when compared with the control individuals. The droughted individuals of Sha showed intermediate phenotypic drought stress-related signs compared with the control batch, such as a minor reduction in leaf rosette size, more wilting of leaves, and a slightly higher decrease of chlorophyll content (Fig. 1A, B). In contrast, the rosette leaves were more reduced in size in the droughted individuals of Col-0, Kel-4, and Cvi compared with the well-watered control plants (Fig. 1A); likewise, leaves and inflorescence stems in the droughted batch of these three genotypes were considerably more wilted compared with the control plants (Fig. 1A), along with the stronger chlorophyll reduction in the rosette leaves (Fig. 1B). With regards to Chl *b* reduction during the drought experiment, two significantly different genotype groups could be defined: one group comprising Col-0, Cvi, and Kel-4 (62, 67, and 46% reduction, respectively) and the other comprising Sha, *soc1ful*, and *p35S:AHL15* (31, 13, and 27% reduction, respectively) (*F=*15.83, *P=*0.00212). For Chl *a* reduction, significant differences were detected among the genotypes (*F=*181.6, *P=*1.84e^−06^), except for *soc1ful* and *p35S:AHL15* that presented a similar reduced value (10% and 12% reduction). This is also the case for total chlorophyll (Chl *a*+*b*) reduction (*F=*168.1, *P=*2.32e^−06^) (Fig. 1B).

In order to estimate how each Arabidopsis genotype senses drought stress at the molecular level, we measured the expression of four selected drought marker genes at the end of the 15–17 d drought treatment. In the ecotypes with an intermediate level of stem lignification (Col-0 and Kel-4) and the one with the least lignified stems (Cvi), all four drought-responsive genes were up-regulated under drought compared with well-watered conditions (Fig. 1C). In contrast, the four drought-response genes in the more lignified genotypes Sha, the overexpression line *p35S:AHL15*, and *soc1ful* were significantly less induced under drought treatment. Interestingly, *p35S:AHL15* showed no difference in *ABI2* and *AREB1* expression level between drought and control conditions (–0.45 and –1.37 log2 fold change, respectively). Regarding the changes in the expression of each gene between drought and control conditions among genotypes studied, we found that the change of *RD29A* expression was similar between Col-0 and Cvi (~6.9 log2 old change). Still, these two genotypes were significantly different from the rest (2.8–4.7 log2 fold change) (*F=*10.2, *P=*0.00021). For *DREB2A*, two significantly different groups were defined: one comprising Col-0, Cvi, and Kel-4 (4.55, 5.6, and 5.57, respectively) and the other comprising Sha, *soc1ful*, and *p35S:AHL15* (3.37, 2.75, and 2.87, respectively) (*F=*21.05, *P=*2.71e^−06^). The changes of *AREB1* were significantly different among genotypes (*F=*13.28, *P=*4.63e^−05^), except for Col-0, Cvi, and Kel-4 (3.48, 3.22 and 3.19 log2 fold change, respectively). Likewise, for *ABI2*, there was a significant difference among genotypes (*F* =40.95, *P=*3.2e^−08^), except for Col-0 and Kel-4 (6.22 and 5.93), and Sha and *soc1ful* (4.57 and 3.58 log2 fold change) (Fig. 1C).

### Leaf water potential (Ψ_l_) and stomatal conductance (*g*_s_) dynamics during drought

**Fig. 2.**
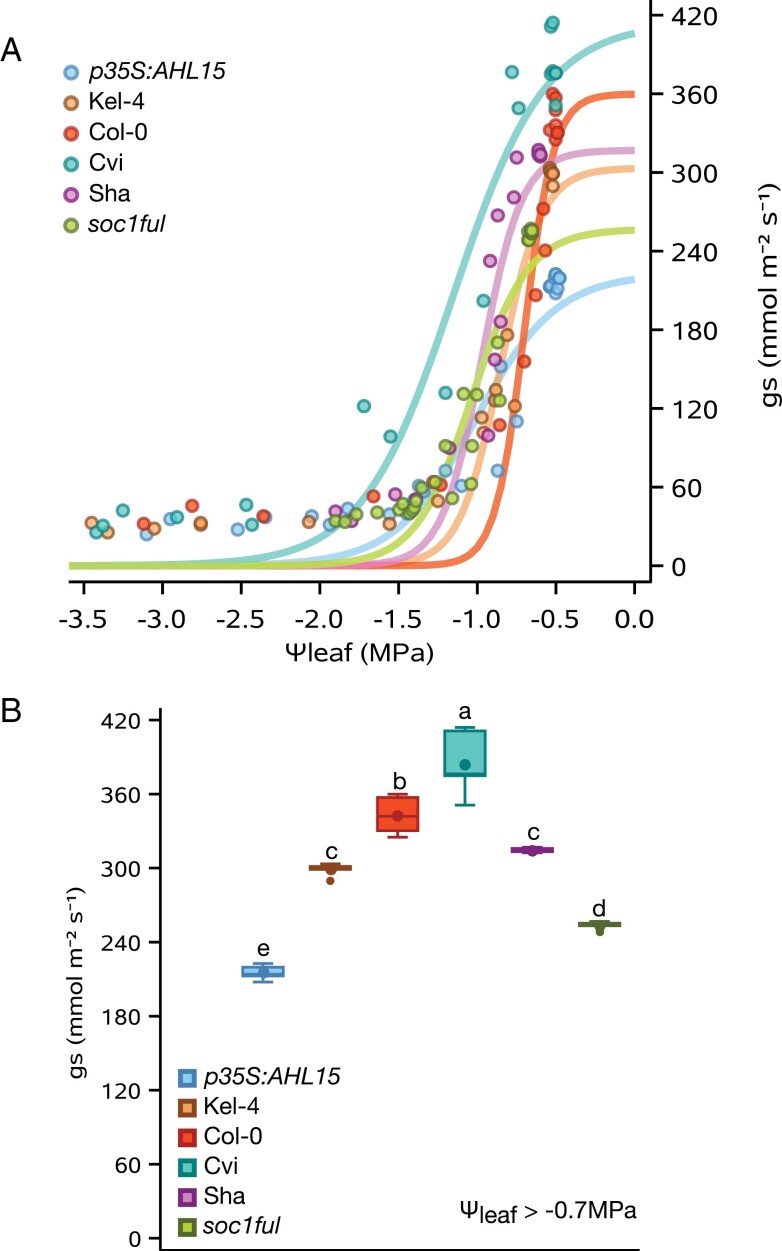
Drought**-**response traits for the six *A. thaliana* genotypes studied. (A) The relationship between leaf water potential (Ψ_l_) and stomatal conductance (*g*_s_). (B) *g*_s_ (mmol s^−1^ m^−2^) in control well-watered plants for the different Arabidopsis genotypes (leaf water potential > –0.7 MPa). Larger symbols within boxes correspond to means, and smaller symbols outside boxes to outlier values. The error bars show the SE based on three biological replications. Colours refer to the genotype studied: Col-0, red; Cvi, turquoise; Sha, purple; *soc1ful*, green; *p35S:AHL15*, blue; Kel-4, brown.

Ψ_l_ under well-watered conditions was similar in every genotype, ranging between –0.5 MPa and –0.6 MPa (Fig. 2A). However, *g*_s_ of control plants was significantly different among the genotypes studied (*F*=236.12, *P<*0.0001, Fig. 2B). Cvi (least lignified wild type) had the highest *g*_s_ (384 mmol m^−2^ s^−1^), followed by Col-0, Sha, and Kel-4, while the more lignified *soc1ful* and *p35S:AHL15* genotypes presented the lowest *g*_s_ value (up to 216 mmol m^−2^ s^−1^); only *g*_s_ values of Sha and Kel-4 were not statistically different from each other (Fig. 2B). In addition, we noticed that Col-0 closed its stomata at a less negative leaf water potential compared with the other genotypes. It reached 90% of stomatal closure (*g*_s90_) at –0.9 MPa, followed by Kel-4 (–1.13 MPa), and the more lignified Sha (–1.27 MPa), *soc1ful* (–1.43 MPa), and *p35S:AHL15* (–1.6 MPa). The least lignified Cvi reached more negative Ψ_l_, even before closing its stomata (–1.75 MPa; Fig. 2A). When following stomatal conductance and leaf water potential decline during the drought experiment, we found that the lignified *soc1ful* and Sha genotypes never reached critical water potential values (i.e. the *P*_50_) even after 17 d of drought, while other genotypes reached their respective *P*_50_ between 10 d and 14 d (Supplementary Fig. S2A, B).

### Stem vulnerability to embolism

**Fig. 3.**
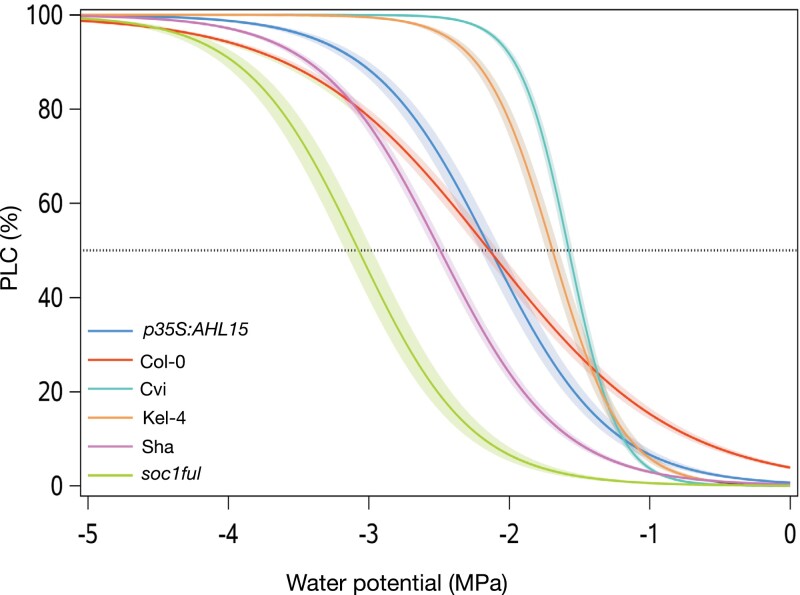
Mean vulnerability curves present the percentage loss of conductivity (PLC) as a function of xylem pressure (MPa) of each genotype studied. Shaded bands represent the SEs based on 5–10 vulnerability curves per genotype. Colours refer to the genotype studied: Col-0, red; Cvi, turquoise; Sha, purple; *soc1ful*, green; *p35S:AHL15*, blue; Kel-4, brown.

When comparing all six genotypes, the most lignified *soc1ful* was the most embolism resistant, with *P*_50_ of –3.07 MPa (Fig. 3; Table 1), whereas the least lignified Cvi remained the most vulnerable (*P*_50_= –1.58 MPa). For the two added genotypes, Kel-4 (wild type with intermediate lignified stems) was among the most vulnerable genotypes with *P*_50_= –1.69 MPa, whereas *p35S:AHL15* (overexpression line) was intermediate, almost identical to the common wild-type Col-0 with *P*_50_= –2.13 MPa. The *P*_12_ (stem water potential at onset of embolism) values of most of the genotypes studied were different from each other (*F*=420.6; *P<*2e^−16^), but Cvi and Kel-4 presented similar *P*_12_ (*P*=0.5424). For *P*_88_, *p35S:AHL15* and Kel-4 were different from other genotypes (*F*=75.09; *P*<2e^−16^) (Supplementary Fig. S3). The slope of the vulnerability curve was similar across the genotypes, except Col-0, which had a lower slope (see Fig. 3).

**Table 1.** The hydraulic data of Arabidopsis genotypes studied measured during the drought experiment

Genotypes	*P*_50_ (MPa)	Ψ*g*_s90_ (MPa)	SSM (MPa)	Ψ_lh_ (MPa)	Days until 90% stomatal closure	Days until *P*_50_	PLC after 3 weeks of non-watering
Cvi	–1.58	–1.75	–0.17	–3.4	10	10	100%
Kel-4	–1.69	–1.13	0.56	–3.4	11	11	100%
Col-0	–2.14	–0.9	1.24	–2.97	10–11	12	75%
*p35S:AHL15*	–2.13	–1.6	0.53	–3.03	13	14	88%
Sha	–2.49	–1.27	1.22	–1.85	12	Does not reach *P*_50_	14%
*soc1ful*	–3.07	–1.43	1.64	–1.87	14	Does not reach *P*_12_	10%

*P*_50_, stem water potential at 50% loss of hydraulic conductivity; Ψ*g*_s90_, leaf water potential at 90% stomatal closure; SSM, stomatal safety margin; Ψ_lh_, leaf water potential at the harvesting day; PLC, percentage loss of hydraulic conductivity.

### Water potential and SSM during drought

**Fig. 4.**
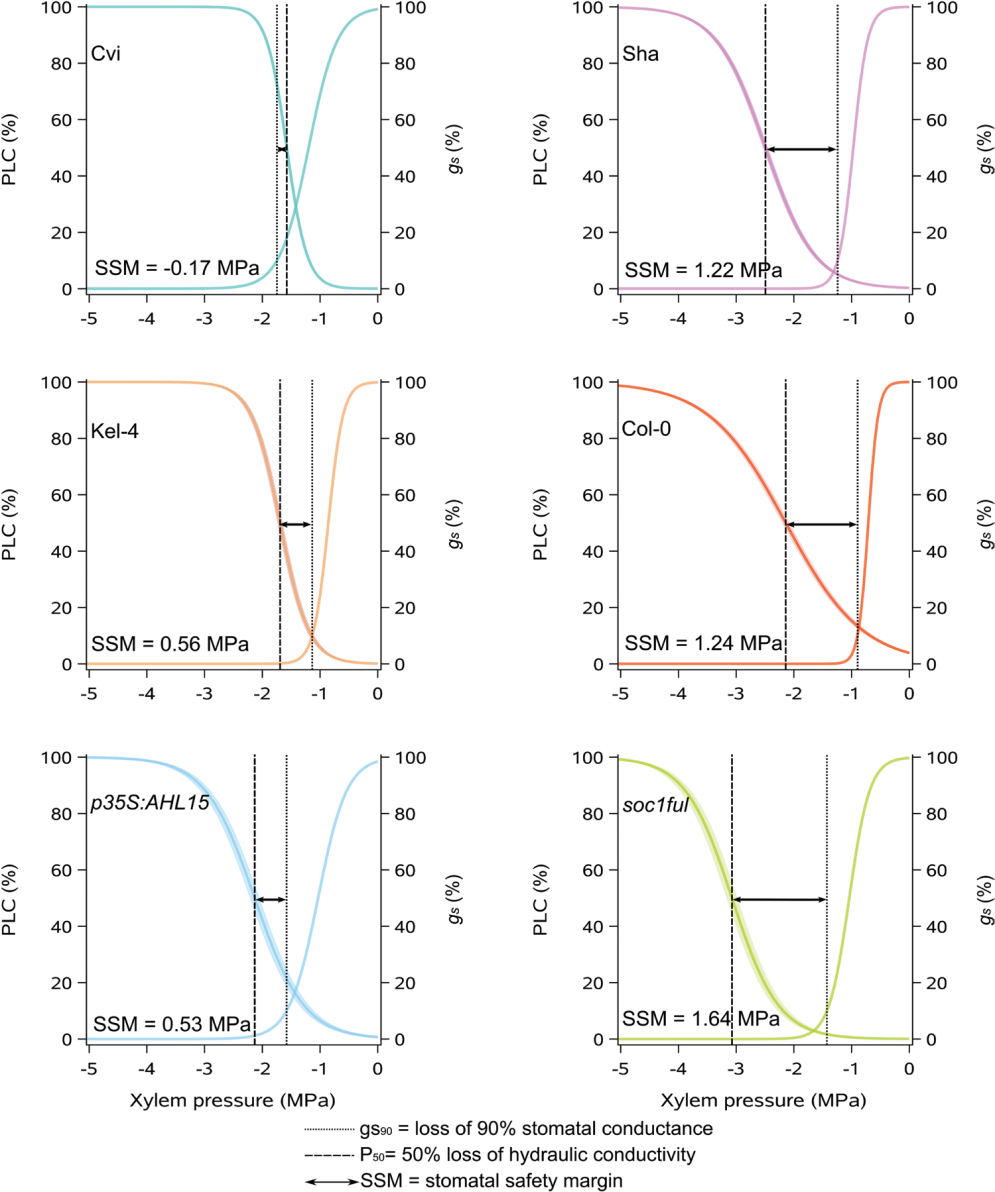
Stomatal safety margin (SSM) of each genotype studied. The graphs show the percentage loss of hydraulic conductivity (PLC) and the percentage of stomatal conductance (*g*_s_) as a function of xylem pressure (MPa). The dotted lines represent water potential at 90% loss of stomatal conductance. The dashed lines show the *P*_50_. The difference between the dashed and the dotted line refers to the SSM.

Assuming that leaf water potential values are similar to stem water potential values in the tiny Arabidopsis herbs, we calculated the SSM as the difference between Ψ*g*_s90_ and *P*_50_. The SSMs of all genotypes studied were positive (from +0.53 MPa to +1.64 MPa), except for the least lignified Cvi with a narrow and negative SSM (–0.17 MPa) (Fig. 4). Accordingly, Cvi also closed its stomata and reached a leaf water potential equivalent to *P*_50_ the soonest (10 d; Table 1). SSM was the widest in the most lignified *soc1ful* (+1.64 MPa), followed by Col-0 and Sha (+1.24 MPa and +1.22 MPa, respectively; Table 1; Fig. 4). Kel-4 and *p35S:AHL15* had intermediate SSMs (+0.56 MPa and +0.53 MPa, respectively).

### The differences in anatomical features among genotypes studied

When comparing the anatomical dataset across the six genotypes, we found that the lignified *soc1ful* and Sha genotypes had the thickest intervessel pit membranes (T_PM_), followed by an intermediate pit membrane thickness of *p35S:AHL15* and Col-0 (*F*=3.857; *P=*0.0672), and thinner pit membranes in Kel-4 and the least lignified Cvi (*F*=4.467; *P=*0.0506) (Supplementary Fig. S4A). Results of vessel wall thickness (T_V_) showed the same pattern as that described for intervessel pit membrane thickness (*F*=2.546; *P=*0.13 and *F*=0.554; *P=*0.468, respectively) (Supplementary Fig. S4B). Vessel grouping index (V_G_) was markedly higher in the *p35S:AHL15* overexpression line than in all the other genotypes (*F*=27.38; *P=*5.46e^−13^) (Supplementary Fig. S4C), which was also the case for the proportion of lignified area per total stem area (P_LIG_; *F*=28.8; *P=*2.25e^−13^) (Supplementary Fig. S4D). The lignified *p35S:AHL15* overexpression line also had a higher proportion of fibre wall area per fibre cell area (PF_W_F_A_) than Kel-4, Col-0, and Cvi, but the fibres were less thick walled compared with the lignified genotypes *soc1ful* and Sha (*F*=49.05; *P*<2e^−16^) (Supplementary Fig. S4E). Surprisingly, *p35S:AHL15* showed no wood formation at the stem segment investigated (Supplementary Fig. S1E) and was less lignified than *soc1ful*, although *AHL15–SOC1–FUL* belong to the same pathway. The vessel diameter (D) of Kel-4 was significantly narrower than that of the other genotypes. Among the remaining genotypes, Cvi (least lignified wild type) had the widest mean D, which was significantly different from the *p35S:AHL15* overexpression line, but there was no statistical difference in D with Col-0, Cvi, Sha, and *soc1ful* (*F*= 9.46; *P*=2.52e^−06^) (Supplementary Fig. S4F). For theoretical vessel implosion resistance (T_VW_/D_MAX_)^2^, the lignified *soc1ful* and Sha showed the highest values as well, while there was no difference among *p35S:AHL15*, Kel-4, Col-0, and Cvi (*F*=3.955; *P=*0.0166). Finally, vessel density (V_D_) of *p35S:AHL15*, Col-0, Cvi, Sha, and *soc1ful* was similar (*F*=1.899; *P=*0.13) and significantly higher than that of Kel-4.

### Stem anatomical traits explaining variation in embolism resistance

**Fig. 5.**
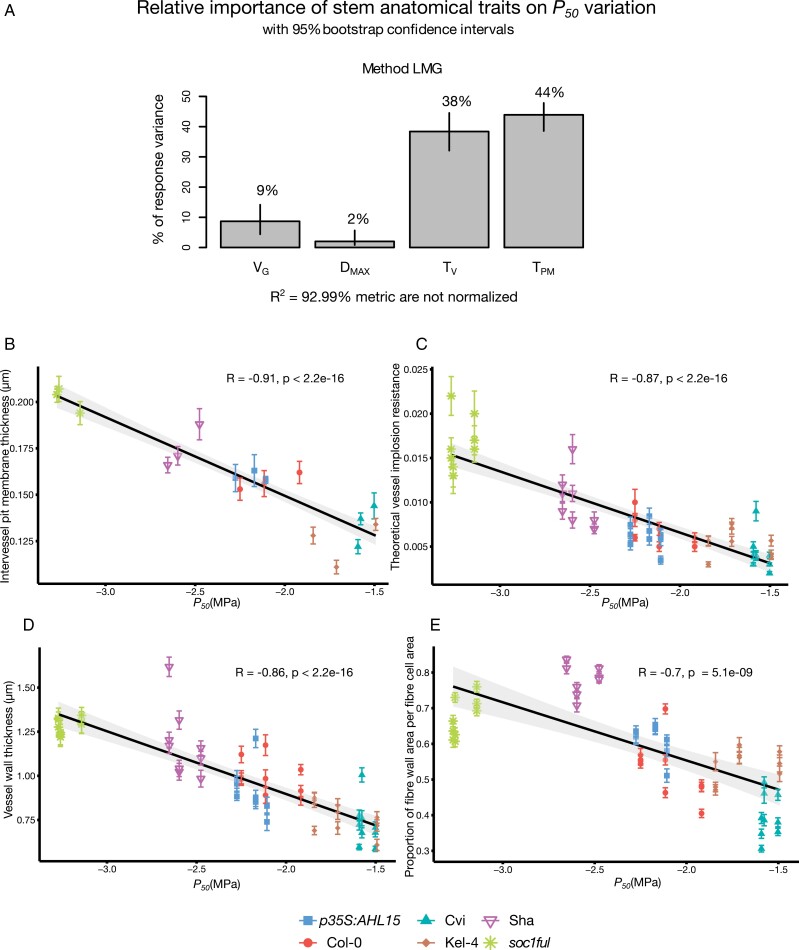
(A) Relative importance of stem anatomical traits on *P*_50_ variation. The *P*_50_ variation is mainly explained by intervessel pit membrane thickness (T_PM_) and vessel wall thickness (T_V_) based on the *R*^2^ contribution averaged over orderings among regressors [based on the Lindemann, Merenda, and Gold (LMG) method]. (B) Negative correlation between T_PM_ and *P*_50_. (C) Negative correlation between (T_VW_/D_MAX_)^2^ and *P*_50_. (D) Negative correlation between T_V_ and *P*_50_. (E) Negative correlation between PF_W_F_A_ and *P*_50_. The error bars show the SEs based on three biological replications for T_PM_ and nine biological replications for other anatomical traits. Colours and styles refer to the genotype studied: Col-0, red circles; Cvi, turquoise upright triangles; Sha, inverted purple triangles *soc1ful*, green stars; *p35S:AHL15*, blue squares; Kel-4, brown diamonds.

According to the most parsimonious model derived from multiple linear regression (AIC= –194.59), the stem anatomical predictors that explain the embolism resistance variation were T_PM_, T_V_, V_G_, and maximum vessel lumen diameter (D_MAX_) (*R*^2^=0.924; *P<*2.2e^−16^) (Supplementary Table S3). T_PM_ was the anatomical feature explaining *P*_50_ variation best, with relative importance of 44%, followed by T_V_ (38%), V_G_ (9%), and D_MAX_ (2%) (Fig. 5A). Likewise, T_PM_ and T_V_ together also explained most of the variation in *P*_12_, with 41% relative importance (*R*^2^=0.795; *P=*1.135e^−14^) (Supplementary Table S4; Supplementary Fig. S5A). *P*_88_ variation, on the other hand, was mostly explained by PF_W_F_A_ (25% relative importance) (*R*^2^=0.516; *P=*1.07e^−07^) (Supplementary Table S5; Supplementary Fig. S5B).

### The relationship among embolism resistance, anatomical traits, and hydraulic traits

Based on a Pearson’s correlation test, T_PM_ was strongly positively correlated with other anatomical traits, such as T_V_, (T_VW_/D_MAX_)^2^, PF_W_F_A_, and V_D_ (*r=*0.77 and *P=*1.108e^−11^; *r=*0.74 and *P=*1.956e^−10^; *r=*0.61 and *P=*8.96e^−07^, *r=*0.58, *P=*4.472e^−06^, respectively) (Supplementary Fig. S6). Lastly, T_V_ and PF_W_F_A_ were correlated as well (*r=*0.71, *P=*2.3e^−09^) (Supplementary Fig. S6). When also taking *P*_50_ into account, we saw that *P*_50_ was strongly correlated with T_PM_, (T_VW_/D_MAX_)^2^, T_V_, and PF_W_F_A_ (*r=* –0.91, –0.87, –0.86, and –0.70; *P*<2.2e^−16^, respectively) (Fig. 5B–E; Supplementary Fig. S6). Similarly, *P*_12_ had strong relationships to T_PM_, (T_VW_/D_MAX_)^2^, and T_V_ (*r=* –0.77 and *P*=6.41e^−12^; *r=* 0.84 and *P*=3.93e^−15^; *r=* 0.68 and *P*=1.38e^−08^, respectively) (Supplementary Fig. S6). *P*_88_ only showed a correlation with PF_W_F_A_ (*r=* –0.54; *P*=2.762e^−05^) and T_V_ (*r=* –0.44; *P*=0.0008146) (Supplementary Fig. S6). We also found a strong correlation between *P*_50_ and the leaf water potential at the harvesting day (Ψ_lh_), the number of days until reaching 90% stomatal closure (Day_90_), and the SSM (*r=* –0.9, –0.85, and –0.84; *P*<2.2e^−16^, respectively), but not between *P*_50_ and Ψ*g*_s90_. Subsequently, the anatomical traits that were strongly correlated to *P*_50_, such as T_PM_, T_V_, and V_G_, were also significantly correlated to Ψ_lh_, Day_90_, and SSM (Supplementary Fig. S6).

## Discussion

We performed a drought experiment including six Arabidopsis genotypes, during which we compiled a detailed xylem anatomical–hydraulic dataset of inflorescence stems (among others intervessel pit membrane thickness, proportion of lignification, and *P*_50_) and leaves (rate of stomatal conductance, leaf water potential, and chlorophyll content), and validated the drought response of the genotypes with the transcript abundance of four known drought marker genes at the end of a 15–17 d treatment without watering. Based on anatomical, hydraulic, and gene expression results, it is clear that the most lignified mutant *soc1ful* (Melzer *et al.*, 2008; Lens *et al.*, 2012, 2013) is the most drought-tolerant genotype, closely followed by the lignified ecotype Sha and the *p35S:AHL15* overexpression line, while the lesser lignified Col-0, Kel-4, and especially Cvi ecotypes are much more sensitive. Interestingly, each genotype applies a unique combination of anatomical stem traits and hydraulic traits in stems and leaves to acquire a certain level of drought tolerance, as will be discussed in the following sections.

### Comparing extremes in drought response: most lignified *soc1ful* versus least lignified Cvi

Both the most drought-tolerant *soc1ful* and the most drought-sensitive Cvi use a similar set of traits with contrasting trait values to reach the two extremes of the drought tolerance spectrum among the genotypes studied. The drought-tolerant strategy of *soc1ful* (Fig. 1A) is determined by a unique combination of traits, as exemplified by the most negative stem *P*_50_ (Fig. 3; cf. Choat *et al.*, 2012; Lens *et al.*, 2016; Thonglim *et al.*, 2020), coupled with a low initial *g*_s_ that gradually slowed down during drought, allowing a more stable leaf water potential (Supplementary Fig. S2A) (Li *et al.*, 2017; Dayer *et al.*, 2020; Lemaire *et al.*, 2021). In addition to its low *g*_s_, *soc1ful* started closing its stomata rapidly at the onset of drought (at high water potential) to further reduce water loss, but at the same time it reached full stomatal closure later than in the other genotypes (Ψ*g*_s90_ was reached after 14 d of non-watering, Table 1). Although we had not quantified carbon uptake during drought, we observed that stomatal closure in *soc1ful* occurred gradually over a longer period during drought, probably extending photosynthetic activities without risking a detrimental level of drought-induced embolism (Fig. 2A; Supplementary Fig. S2). This is further supported by a low reduction of chlorophyll content in rosette leaves of droughted *soc1ful* individuals compared with the well-watered control batch (Fig. 1B), Moreover, this mutant line had the widest positive SSM (Fig. 4), which is essential in estimating a plant’s drought response (Choat *et al.*, 2012; Delzon and Cochard, 2014; Anderegg *et al.*, 2016; Eller *et al.*, 2018; Oliveira *et al.*, 2021; Skelton *et al.*, 2021). Finally, as reported in Thonglim *et al.* (2020), this mutant also produced the thickest intervessel pit membranes and largest wood cylinder at the base of the inflorescence stem. Both traits are thought to play an important role in preventing embolism spread (Lens *et al.*, 2022). In contrast, the least lignified Cvi was the most vulnerable genotype as it showed the least negative stem *P*_50_ combined with a rapid drop in leaf water potential during drought, leading to rapid wilting (Fig. 1A) and a strong decrease of chlorophyll content (Fig. 1B). In addition, Cvi had the highest initial *g*_s_, and it closed its stomata at low water potential, which led to more water loss due to transpiration (Fig. 2A; Supplementary Fig. S2). Although it reached Ψ*g*_s90_ earlier than the more tolerant genotypes (Table 1), it seemed like Cvi could not close its stomata in time because all the water was already consumed, giving rise to a rapid water potential drop during drought (Supplementary Fig. S2A). Due to its less negative stem *P*_50_, the Ψ*g*_s90_ exceeded stem *P*_50_, leading to the only negative SSM among the six genotypes studied (Fig. 4). This implies that Cvi experiences a considerable decrease in stem hydraulic conductivity right after or even before stomatal closure. In addition to all these physiological parameters pointing to the most sensitive drought response among the genotypes studied, Cvi also had the least lignified inflorescence stems with the thinnest intervessel pit membranes (Thonglim *et al.*, 2020).

### The role of embolism resistance and stomatal regulation in drought tolerance and its impact on the stomatal safety margin

The previous section highlights the importance of embolism resistance as well as SSMs in determining drought tolerance, as has been demonstrated across many other lineages of plants (Meinzer *et al.*, 2009; McDowell, 2011; Choat *et al.*, 2012; Johnson *et al.*, 2012; Cochard *et al.*, 2013; Lens *et al.*, 2013; Skelton *et al.*, 2015, 2021; Martin-StPaul *et al.*, 2017; Creek *et al.*, 2020; Dayer *et al.*, 2020). However, our dataset suggests that stem *P*_50_*—*which is probably a good proxy for whole-plant *P*_50_ based on our few leaf *P*_50_ measurements in the *p35S:AHL15* overexpression line and based on other herbaceous species showing no difference in *P*_50_ across organs (e.g. Skelton *et al.*, 2017)—outperforms SSM in explaining the responses to drought among the genotypes studied. This is because stomatal regulation in Arabidopsis genotypes that were equally drought tolerant could be substantially different, while *P*_50_ showed a more consistent pattern with whole-plant drought tolerance. However, it seems that the rate of *g*_s_ in Arabidopsis under well-watered conditions is more critical than the speed of stomatal closure, as shown by Cvi, Col-0, and Kel-4 (Table 1; Supplementary Fig. S2B). Indeed, Ψ*g*_s90_ is not the driving force behind drought tolerance since the more drought-tolerant genotypes closed their stomata slightly later than the sensitive ones. In other words, Cvi, Col-0, and Kel-4 lost more water because of a higher transpiration rate, but they closed their stomata sooner than the more drought-tolerant genotypes (Table 1). These results align with previous studies stating that stomatal behaviour only shows how each species respond to drought stress, but not how much they tolerate drought (Roman *et al.*, 2015; Combe *et al.*, 2016; Martínez-Vilalta and Garcia-Forner, 2017). Bearing this in mind, our observation shows that the two mutant genotypes studied in the Col-0 background (*soc1ful* and *p35S:AHL15*)—both belonging to the same regulatory SOC1–FUL–AHL15–cytokinin pathway that induces wood formation in stems (Rahimi *et al.*, 2022)—also have by far the lowest initial *g*_s_ values across all six genotypes studied, including the Col-0 ecotype (Fig. 2B). This makes it a promising gene regulatory pathway to discover how drought-responsive traits in stems (increased lignification or woodiness) and leaves (reduced *g*_s_) are linked to each other at the genetic level.

Our dataset aligns with earlier studies showing that safety margins across (mainly woody) angiosperms are overall positive, and considerable levels of embolisms only happen under remarkable, intense drought events (Choat *et al.*, 2012; Delzon and Cochard, 2014; Martin-StPaul *et al.*, 2017; Creek *et al.*, 2020; Dayer *et al.*, 2020; Skelton *et al.*, 2021; Guan *et al.*, 2022; Lens *et al.*, 2022). The positive SSMs in five out of six genotypes indicate that stomatal closure typically occurs before embolism in order to prevent water loss and delay hydraulic dysfunction (Martin-StPaul *et al.*, 2017; Creek *et al.*, 2020). In contrast, Cvi—the only genotype with a negative SSM—closed its stomata at 70% loss of maximum conductance, highlighting its high sensibility to drought.

### Multiple strategies to acquire drought tolerance

In addition to the drought-responsive traits discussed in *soc1ful* and Cvi, different combinations among these traits were observed in the remaining genotypes. This shows that even in a species with a short life cycle, multiple strategies can be applied to acquire a certain level of drought tolerance. For instance, Sha and *p35S:AHL15* had a similarly high level of drought tolerance based on their phenotype after 3 weeks of water shortage (Fig. 1A), but their drought-responsive traits were different. Sha had high embolism resistance in stems combined with a relatively high initial transpiration rate in leaves that rapidly declines during drought, allowing a relatively stable leaf water potential (also confirmed by Bouchabke *et al.*, 2008) and a large SSM. On the other hand, *p35S:AHL15* had the lowest *g*_s_ of all the genotypes studied (Fig. 2A), which means it can keep its leaf water potential relatively high during drought, whereas its stem *P*_50_ was intermediate and led to a smaller SSM compared with Sha (Figs 2–4). Another example is given by *p35S:AHL15* (overexpression line) and Col-0 common wild type, which both had a similar stem *P*_50_ (–2.1 MPa; Fig. 3). However, Col-0 was more drought sensitive than *p35S:AHL15*, even though the former closed its stomata earlier during drought, resulting in a wider SSM (Fig. 4). The reason for Col-0 being more drought sensitive is that stomatal conductance is much higher, leading to more water loss and consequently a more rapid decline in leaf water potential during the drought experiment, while the leaf water potential during drought in *p35S:AHL15* drops more slowly (Supplementary Fig. S2). Thus, a wider SSM does not always lead to a prolonged survival during drought since the rate of *g*_s_ is not accounted for in the SSM. In other words, the width of the safety margin does not necessarily match all aspects of stomatal regulation and the resulting leaf water potential dynamics during drought (Martínez-Vilalta and Garcia-Forner, 2017; Martin-StPaul *et al.*, 2017; Knipfer *et al.*, 2020).

### Expression levels of drought-responsive genes agree with drought-response traits

To assess the level of drought stress and compare it among the genotypes, we assessed the expression of selected drought-responsive genes on the final day of the drought treatment (15–17 d). As expected, the four drought-responsive genes *RD29A*, *DREB2A*, *ABI2*, and *AREB1* were most up-regulated in the more sensitive genotypes Col-0, Kel-4, and Cvi, and less up-regulated in the more tolerant genotypes Sha, *p35S:AHL15*, and *soc1ful* (Fig. 1C). To study the casual relationship between physiological responses (e.g. stomatal closure) and gene activity (e.g. ABA biosynthesis genes), future work should focus on conducting a high-resolution time-course gene expression analysis, which is beyond the scope of this study.

### Intervessel pit membrane thickness as an important anatomical driver of embolism resistance, and the potential effect of stem lignification on *P*_50_

Our extended database confirms our previous results that intervessel pit membrane thickness is the anatomical trait that explains best the variation in *P*_50_ across all six genotypes studied (Fig. 5A). These results are in line with several other angiosperm studies showing a strong positive correlation between embolism resistance and T_PM_, both at the interspecies level (Jansen *et al.*, 2009; Lens *et al.*, 2011, 2022; Plavcová and Hacke, 2012; Plavcová *et al.*, 2013; Scholz *et al.*, 2013b; Li *et al.*, 2016; Dória *et al.*, 2018; Trueba *et al.*, 2019; Guan *et al.*, 2022) and within species (Schuldt *et al.*, 2016). The functional explanation for this relationship was intensively discussed in our previous paper (Thonglim *et al.*, 2020). In brief, there is convincing evidence based on microCT and/or optical technique observations in stems (Brodersen *et al.*, 2013; Knipfer *et al.*, 2015; Choat *et al.*, 2016; Skelton *et al.*, 2017; Torres-Ruiz *et al.*, 2017) and leaves (Brodribb *et al.*, 2016; Skelton *et al.*, 2017, 2018; Klepsch *et al.*, 2018; Lamarque *et al.*, 2018) that embolism spread between adjacent vessels predominantly happens via porous pit membranes located inside the bordered pits between adjacent vessels. Although this explains why the thickness of intervessel pit membrane plays an important role in embolism propagation and, by extension, also whole-plant drought tolerance, the detailed mechanisms behind this embolism spread remain poorly known due to the complex 3D structure/composition of pit membranes and the enigmatic behaviour of gas–liquid–solid–surfactant interfaces at the nano-scale (Kaack *et al.*, 2019, 2021; Yang *et al.*, 2020; Zhang *et al.*, 2020; Lens *et al.*, 2022).

It has also been shown in previous studies that intervessel pit membrane thickness is strongly linked not only with *P*_50_, but also with other anatomical traits assumed to be involved in drought-induced embolism resistance, such as vessel wall thickness (Jansen *et al.*, 2009; Li *et al.*, 2016), and the amount of stem lignification or woodiness (Li *et al.*, 2016; Dória *et al.*, 2018; Thonglim *et al.*, 2020). How exactly lignification would impact embolism spread in stems is the subject of ongoing research. One hypothesis is that the amount of lignification in secondary cell walls may determine gas diffusion kinetics across xylem cell walls and, therefore, could reduce the speed of embolism propagation in species with increased levels of lignification or woodiness (Li *et al.*, 2016; Dória *et al.*, 2018; Pereira *et al.*, 2018; Thonglim *et al.*, 2020; Lens *et al.*, 2022). This may imply that older stems from herbaceous species could lead to increased embolism resistance, resulting from a possible increase in stem lignification and/or the amount of wood. In our study, this may especially apply to the *p35S:AHL15* overexpression line, which has the ability to develop as much wood as the *soc1ful* double knockout genotype (Rahimi *et al.*, 2022). However, this study shows that wood development is delayed in *p35S:AHL15* (Supplementary Fig. S1E, G) compared with *soc1ful* in 80-day-old plants, despite the fact that SOC1, FUL, and AHL15 belong to the same wood pathway (Rahimi *et al.*, 2022). Older individuals of *p35S:AHL15* will therefore develop more wood and probably also thicker intervessel pit membranes in their inflorescence stems, most probably resulting in both higher embolism resistance and higher SSM, which synergistically may increase total plant tolerance of the overexpression line to the level of *soc1ful*.

In conclusion, there is a considerable difference in drought response among the six Arabidopsis genotypes studied. The genotypes *soc1ful*, Sha, and *p35S:AHL15* synergistically increase their drought tolerance by building lignified inflorescence stems with thick intervessel pit membranes, developing the largest SSMs, keeping the water potential in their leaves pretty stable during periods of water shortage as a result of low stomatal conductance, maintaining relatively high chlorophyll content in rosette leaves, and by showing the lowest expression levels of drought-response genes compared with the control batch. In contrast, the most sensitive genotypes to drought (Cvi, Kel-4, and Col-0) are more susceptible to drought due to the opposite extreme of the same set of drought-responsive traits. This shows that stem anatomical traits and hydraulic stem and leaf traits are intertwined to acquire a certain level of drought tolerance. To further disentangle gene regulatory networks underlying drought-responsive traits across organs and to find out how they are linked with each other and synergistically strengthen the whole-plant drought response, future studies should combine a time series of gene expression data in roots, stems, and leaves during a drought experiment followed by rewatering. During such an experiment, a range of drought-responsive (anatomical and physiological) traits in all organs should be investigated. Only with this integrative approach, will we be able to make considerable progress in securing our food production by developing breeding tools that can make crops more drought tolerant and propose solutions on how to protect our herbs and forests under the current global change scenario.

## Supplementary data

The following supplementary data are available at *JXB* online.

Table S1. Oligonucleotide sequences.

Table S2. The anatomical characters and hydraulic values measured with acronyms, definitions, calculations, microscope techniques, and units.

Table S3. The most parsimonious multiple linear regression model (based on AIC scores) of anatomical traits, explaining stem *P*_50_ variation of the six *Arabidopsis thaliana* genotypes studied.

Table S4. The most parsimonious multiple linear regression model (based on AIC scores) of anatomical traits explaining stem *P*_12_ variation of the six *Arabidopsis thaliana* genotypes studied.

Table S5. The most parsimonious multiple linear regression model (based on AIC scores) of anatomical traits explaining stem *P*_88_ variation of the six *Arabidopsis thaliana* genotypes studied.

Fig. S1. Growth form and cross-sections of inflorescence stems of *p35S:AHL15* and Kel-4. Fig. S2. Leaf water potential and stomatal conductance dynamics during the drought experiment for each genotype.

Fig. S3. Boxplots showing *P*_88_ and *P*_12_ variation within and between genotypes.

Fig. S4. Boxplots showing anatomical variation within and between all genotypes.

Fig. S5. The relative importance of stem anatomical traits with respect to *P*_12_ and *P*_88_.

Fig. S6. The pairwise scatter plots based on Pearson’s correlation analysis showing the correlations of *P*_50_, *P*_12_, and *P*_88_ and each stem anatomical and hydraulic trait studied, and between all the predictive variables.

erac446_suppl_Supplementary_Material

## Data Availability

All data supporting the findings of this study are available within the paper and its supplementary data published online.

## Abbreviations

D: mean diameter of vessels
Day_90_: days until reaching 90% of stomatal closure
D_MAX_: maximum vessel lumen diameter
*g*_s_: stomatal conductance
*P*_12_: xylem pressure inducing 12% loss of hydraulic conductivity
*P*_50_: xylem pressure inducing 50% loss of hydraulic conductivity
*P*_88_: xylem pressure inducing 88% loss of hydraulic conductivity
PF_W_F_A_: proportion of fibre wall area per fibre cell area
P_LIG_: proportion of lignified area per total stem area
SSM: stomatal safety margin
T_PM_: intervessel pit membrane thickness
T_V_: vessel wall thickness
(T_VW_/D_MAX_)^2^: theoretical vessel implosion resistance
V_D_: vessel density
V_G_: vessel grouping index
Ψ*g*_s90_: leaf water potential at 90% loss of stomatal conductance
Ψ_l_: leaf water potential
Ψ_lh_: leaf water potential at the harvesting day.
