# Supplementary material for: Drought response in Arabidopsis displays synergistic coordination between stems and leaves
Source: J Exp Bot. 2022 Nov 9;74(3):1004–21. doi: 10.1093/jxb/erac446 (PMC9899417; doi:10.1093/jxb/erac446)
Supplement: erac446_suppl_Supplementary_Material [file erac446_suppl_supplementary_material.pdf]

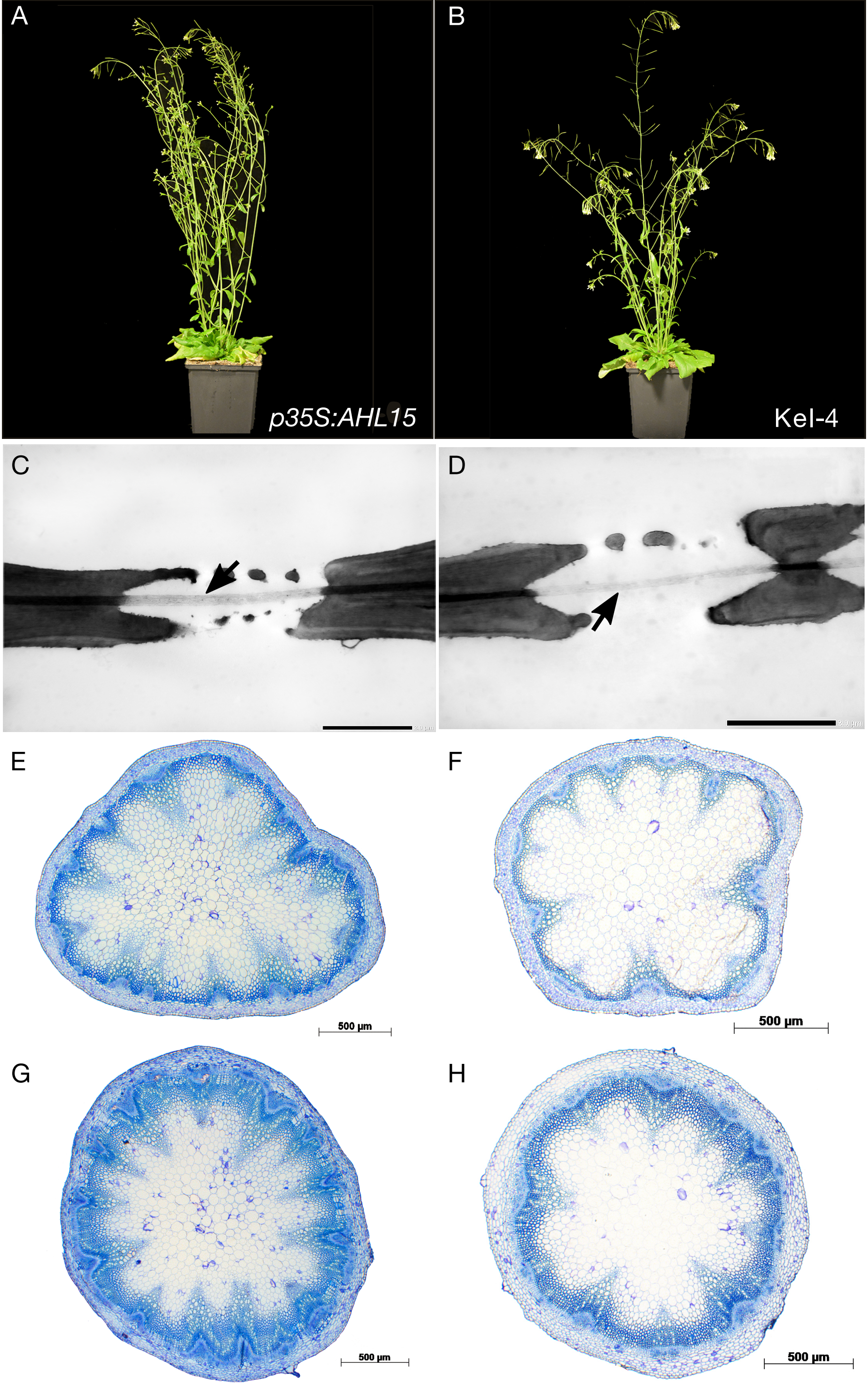

Fig. S1 Growth form and cross-sections of inflorescence stems of *p35S:AHL15* (left, 85d after sowing) and Kel-4 (right, 65d after sowing). (A, B) Growth form. (C, D) TEM images of intervessel pit membranes (arrows). Scale bars = 2 µm. (E, F) LM images of cross-sections at the middle part of inflorescence stems. Scale bars = 500 µm. (G, H) LM images of cross-sections at the basal part of inflorescence stems show more pronounced lignification. Scale bars = 500 µm.

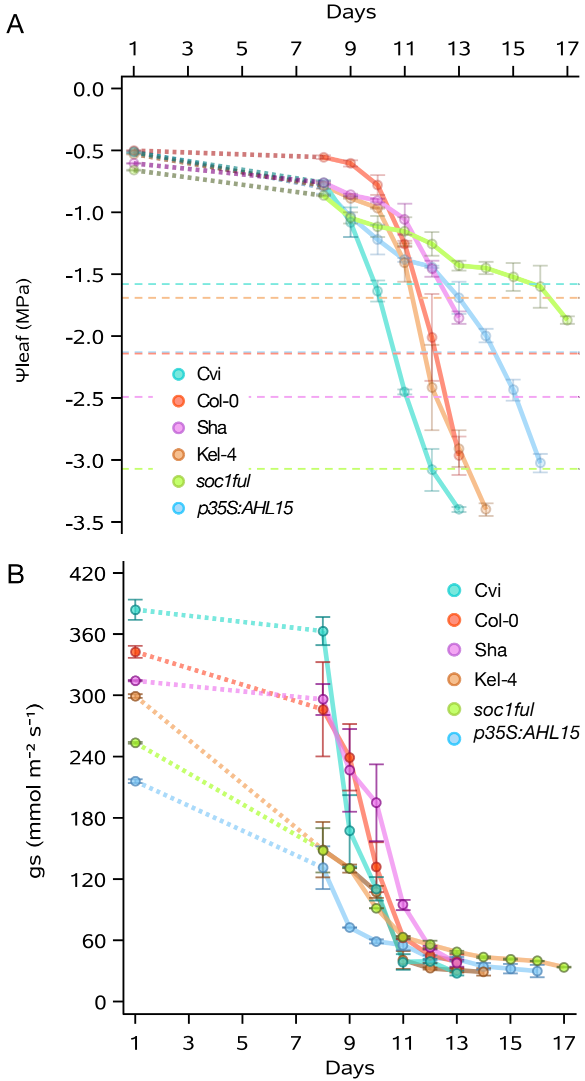

Fig. S2 Leaf water potential and stomatal conductance dynamics during the drought experiment for each genotype. (A) Leaf water potential (Ψ_l)over time. Dotted lines represent P_{50} value of each genotype. (B) Stomatal conductance (g_s) over time. The error bars show standard errors based on three replications. Colours refer to the genotype studied: Col-0, red; Cvi, turquoise; Sha, purple; *soc1ful*, green; *p35S:AHL15*, blue; Kel-4, brown.

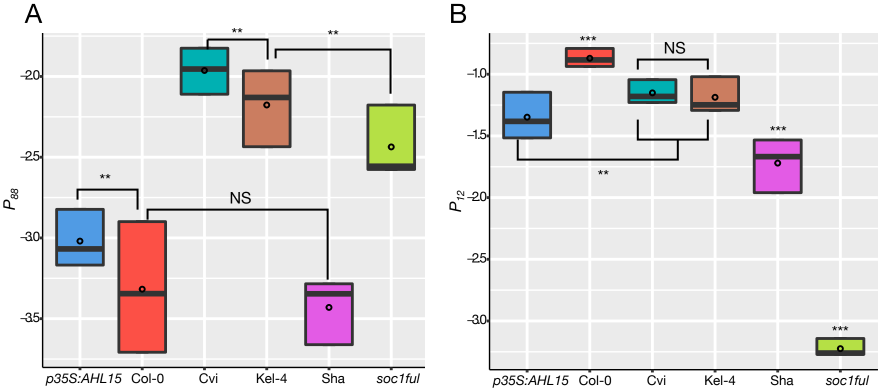

Fig. S3 Boxplots showing P_{88} and P_{12} variation within and between genotypes. (A) Boxplot showing P_{88} of every genotype studied. (B) Boxplot showing P_{12} of every genotype studied. A Newman–Keuls post-hoc test was performed, showing the differences between each genotype; ns = p-value > 0.05; ** p-value < 0.05; *** p-value < 0.01.

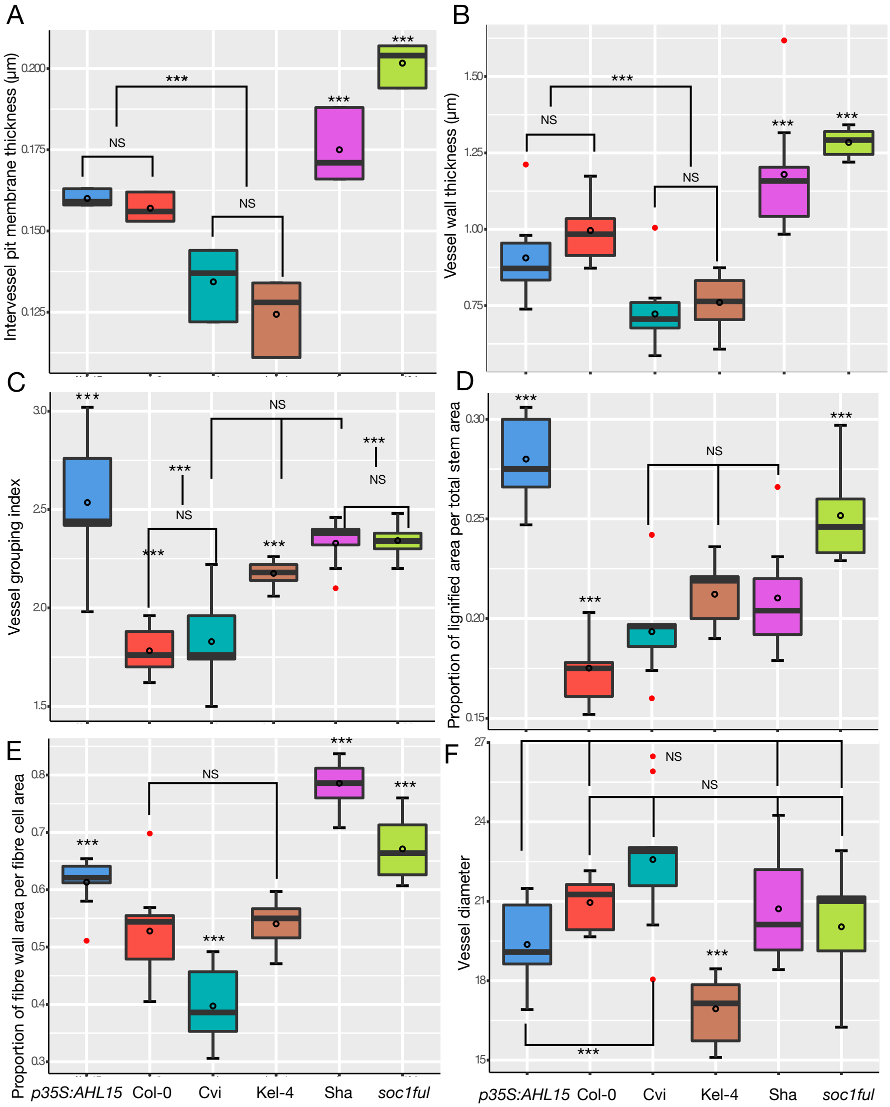

Fig. S4 Boxplots showing anatomical variation within and between all genotypes. (A) Boxplot of intervessel pit membrane thickness (T_{PM}). (B) Boxplot of vessel wall thickness (T_V). (C) Boxplot of vessel grouping index (V_G). (D) Boxplot of the proportion of lignified area per total stem area (P_{LIG}). (E) Boxplot of the proportion of fibre wall area per fibre cell area (PF_WF_A). (F) Boxplot of vessel diameter (D). A Newman–Keuls post-hoc test was performed, showing the differences between each genotype. The error bars show standard errors based on three replications for T_{PM} and nine replications for other anatomical traits; ns = p-value > 0.05; ** p-value < 0.05; *** p-value < 0.01.

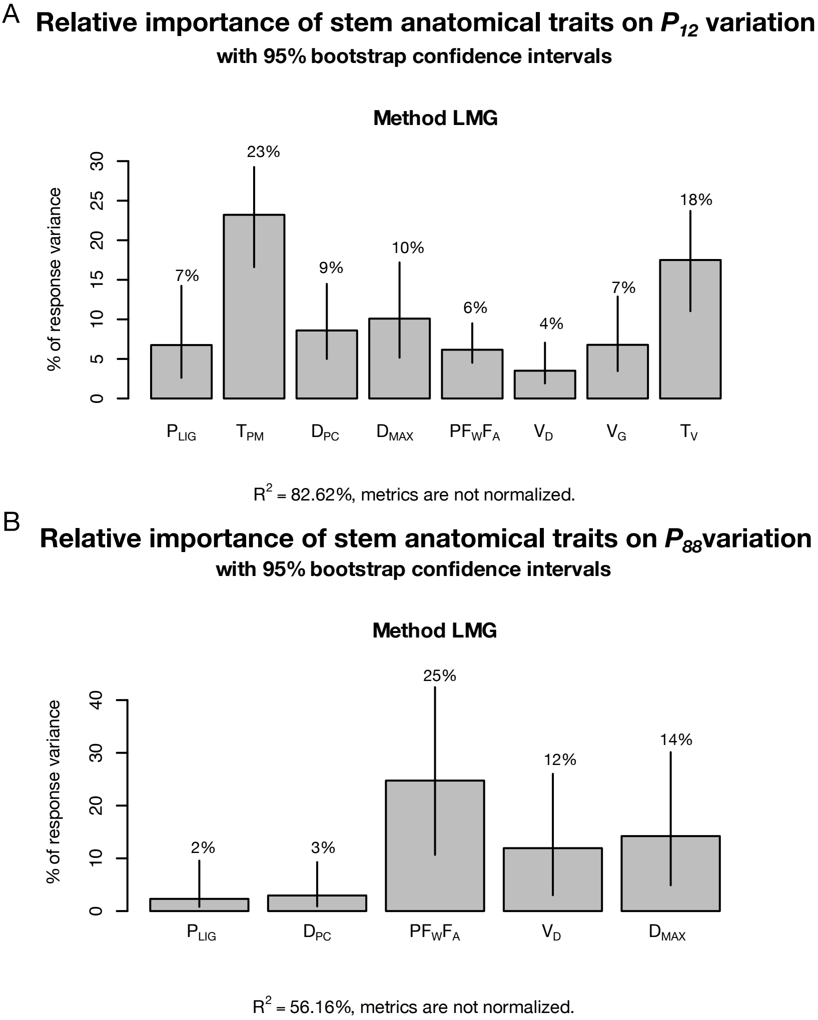

Fig. S5 The relative importance of stem anatomical traits with respect to P_{12} and P_{88}. (A) The relative importance of stem traits on P_{12} variation is mainly explained by intervessel pit membrane thickness (T_{PM}) and vessel wall thickness (T_V). (B) The relative importance of stem traits on P_{88} variation is mainly explained by proportion of fibre wall area per fibre cell area (PF_WF_A).

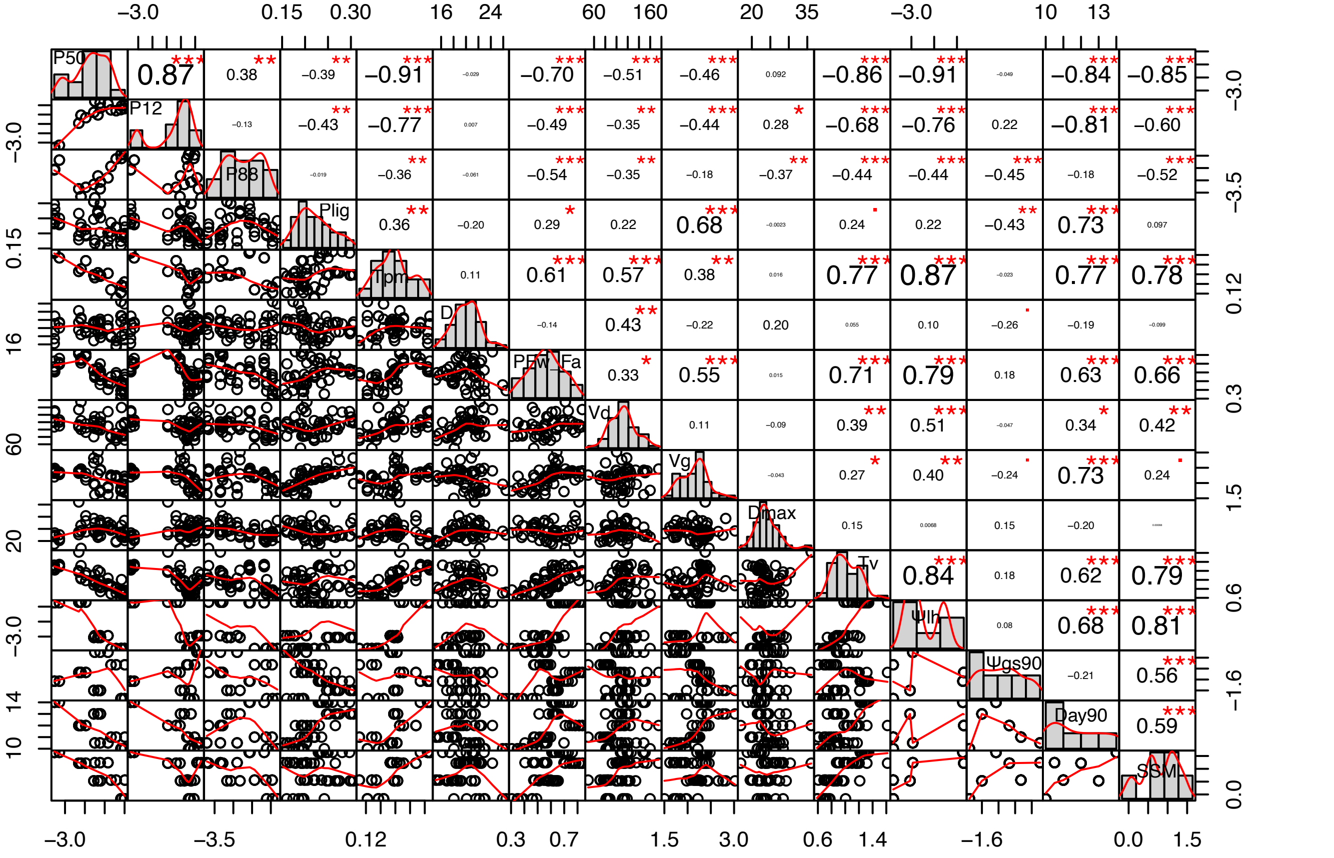

Fig. S6 The pairwise scatter plots based on Pearson's correlation analysis show the correlations of P_{50}, P_{12} and P_{88} (response variables) and each stem anatomical and hydraulic traits studied (predictive variables) and between all the predictive variables. *** p-value < 0.01; ** p-value<0.01; * p-value < 0.05.

Supplementary Table S1 Oligonucleotide sequences.

Primers for qRT-PCR			
**Gene name**	**Gene ID**	**Primer Fwd sequence (5′-3′)**	**Primer Rev sequence (5′-3′)**
ACTIN2	AT3G18780	TCCCTCAGCACATTCCAGCAGAT	AACGATTCCTGGACCTGCCTCATC
GAPDH	At1G13440	TTGGTGACAACAGGTCAAGCA	AAACTTGTCGCTCAATGCAATC
RD29A	AT5G52310	TGGACAAAGCAATGAGCATGAGC	AGGTTTACCTGTTACGCCTGGTG
ABI2	ATG557050	CTCGCAATGTCAAGATCCATTGGC	TTACTCGCCGCACTGAAGTCAC
AREB1	AT1G45249	AGTTACAACGAAAGCAGGCAAGG	CCTCCTTGCAGAAGATTCCTCATC
DREB2A	AT5G05410	CAGTGTTGCCAACGGTTCAT	AAACGGAGGTATTCCGTAGTTGAG

Supplementary Table S2 The anatomical characters and hydraulic values measured with acronyms, definitions, calculations, units, and microscope techniques

Acronyms	Definition	Calculation	Number of measurements	Unit	Technique
A_F	Fibre cell area	Area of single xylem fibre in cross-section	Min. 30 fibres	µm^2	LM
A_{FL}	Fibre lumen area	Area of single xylem fibre lumen in cross-section	Min. 30 fibres	µm^2	LM
A_{FW}	Fibre wall area	A_F minus A_{FL} for the same fibre	Min. 30 fibres	µm^2	LM
A_{LIG}	Lignified stem area	Total xylem area + fibre caps area + lignified pith cell area in cross-section	9 stems per accession	mm^2	LM
A_{PITH}	Pith area	Total pith area in cross section	9 stems per accession	mm^2	LM
A_S	Total stem area	Total stem area in cross section	9 stems per accession	mm^2	LM
D	Diameter of vessels	$D = (\sqrt{4A}/\pi)$	Min. 50 vessels	µm	LM
Day90	Days until reaching 90% of stomatal closure	-	-	days	-

D_{MAX}	Maximum vessel lumen diameter	Diameter of the widest vessel	Min. 30 vessels	µm	LM
D_{PC}	Pit chamber depth	Distance between the relaxed pit membrane and the inner pit aperture	Min. 25 pits	µm	TEM
g_s	Stomatal conductance	$g_s = \frac{\hat{\rho} D_{vapour}}{\frac{[e_s(T_{a1})(1-h_{r1})]d_2}{h_{r1}e_s(T_{a1})-h_{r2}e_s(T_{a2})} - d_1}$	1 control sample and 2 drought samples each measurement	mmol $m^{-2}s^{-1}$	Porometer
SSM	Stomatal safety margin	$\Psi gs_{90} - P_{50}$	1 SSM per accession	MPa	-
P_{12}	Stem water potential at 12% loss of hydraulic conductivity	-	8 values per each accession	MPa	Cavitron centrifuge
P_{50}	Stem water potential at 50% loss of hydraulic conductivity	-	8 values per each accession	MPa	Cavitron centrifuge

P_{88}	Stem water potential at 88% loss of hydraulic conductivity	-	8 values per each accession	MPa	Cavitron centrifuge
PF_WF_A	Proportion of fibre wall area per fibre cell area	A_{FW}/A_F for the same fibre; a measure of xylem fibre wall thickness	Min. 30 fibres	-	LM
Ψgs_{90}	Leaf water potential at 90% loss of stomatal conductance	-	1 control sample and 2 drought samples each measurement	MPa	PSYPRO meter
Ψ_{lh}	Leaf water potential at the harvesting day	-	1 control sample and 2 drought samples each measurement	MPa	PSYPRO meter
P_{LIG}	Proportion of lignified area per total stem area	A_{LIG}/A_S	9 stems per accession	-	LM
T_{PM}	Intervessel pit membrane thickness	Thickness of intervessel pit membrane measured at its thickest point	Min. 25 pit membranes	µm	TEM

T_V	Vessel wall thickness	Thickness of a single vessel wall	Min. 30 vessels	µm	LM
T_{VW}/D_{MAX}	Thickness-to-span ratio of vessels	Double intervessel wall thickness divided by the maximum diameter of the largest vessel	Min. 30 measurements	µm	LM
$(T_{VW}/D_{MAX})^2$	Theoretical vessel implosion resistance	$(T_{VW}/D_{MAX})^2$	Min. 30 measurements	-	LM
V_D	Vessel density	Number of vessels per mm^2	Min. 5 measurements	No. of vessels/ mm^2	LM
V_G	Vessel grouping index	Ratio of total number of vessels to total number of vessel groupings (incl. solitary and grouped vessels)	Min. 50 vessel groups	-	LM

Supplementary Table S3 The most parsimonious multiple linear regression model (based on AIC scores) of anatomical traits, explaining stem P_{50} variation of the six *Arabidopsis thaliana* accessions studied.

Predictors	Estimate	Std. Error	t value	Pr (>|t|)
**(Intercept)**	0.435	0.239	1.825	0.074
**T_{PM}**	-11.096	1.329	-8.347	$5.67e^{-11}$***
**D_{MAX}**	0.028	0.007	4.149	0.000132***
**V_G**	-0.242	0.073	-3.331	0.001651**
**T_V**	-1.074	0.146	-7.360	$1.84\ e^{-09}$**

T_{PM} = intervessel pit membrane thickness; D_{MAX} = maximum vessel lumen diameter; V_G = vessel grouping index; T_V = vessel wall thickness. *** *p-value* < 0.001;
** *p-value* < 0.01

Supplementary Table S4 The most parsimonious multiple linear regression model (based on AIC scores) of anatomical traits explaining stem P_{12} variation of the six *Arabidopsis thaliana* accessions studied.

Predictors	Estimate	Std. Error	t value	Pr (>|t|)
**(Intercept)**	-0.599	0.687	-0.872	0.388
**P_{LIG}**	-3.365	1.793	-1.877	0.067
**T_{PM}**	-12.657	3.660	-3.458	0.001**
**D_{PC}**	1.926	0.756	2.546	0.014*
**D_{MAX}**	0.086	0.016	5.492	$1.76e^{-06}$***
**PF_WF_A**	1.093	0.660	1.656	0.105
**V_D**	0.006	0.003	2.443	0.019*
**V_G**	-0.511	0.261	-1.959	0.056
**T_V**	-1.416	0.404	-3.502	0.001**

P_{LIG} = proportion of lignified area per total stem area; T_{PM} = intervessel pit membrane thickness; D_{PC} = pit chamber depth; D_{MAX} = maximum vessel lumen diameter; PF_WF_A = proportion of fibre wall area per fibre cell area; V_D = vessel density V_G = vessel grouping index; T_V = vessel wall thickness. *** *p-value* < 0.001; ** *p-value* < 0.01; * *p-value* < 0.05

Supplementary Table S5 The most parsimonious multiple linear regression model (based on AIC scores) of anatomical traits explaining stem P_{88} variation of the six *Arabidopsis thaliana* accessions studied.

**Predictors**	**Estimate**	**Std. Error**	**t value**	**Pr (>|t|)**
**(Intercept)**	1.414	0.701	2.018	0.049*
**P_{LIG}**	3.967	1.556	2.550	0.014*
**D_{PC}**	-1.932	0.726	-2.662	0.011*
**PF_WF_A**	-2.208	0.469	-4.710	$2.148e^{-05}$***
**V_D**	-0.010	0.003	-3.575	0.000810***
**D_{MAX}**	-0.072	0.018	-4.054	0.000184***

P_{LIG} = proportion of lignified area per total stem area; D_{PC} = pit chamber depth; PF_WF_A = Proportion of fibre wall area per fibre cell area; V_D = vessel density; D_{MAX} = maximum vessel lumen diameter. *** *p-value* < 0.001; ** *p-value* < 0.01; * *p-value* < 0.05
